# RGB-Based Non-Destructive Measurement of Greenhouse Tomato Fruit Traits Using High-Fidelity Segmentation

**DOI:** 10.3390/plants15192952

**Published:** 2026-09-28

**Authors:** Ke Zhang, Aoxue Shen, Ruocheng Gao, Hong Cheng, Jie Zhu, Guan Pang, Yu Wang, Xia Yao, Pei Zhang, Jin Sun

**Affiliations:** 1 College of Horticulture, Nanjing Agricultural University, Nanjing 210095, China; njauzk@163.com (A.S.); 2017201080@njau.edu.cn (R.G.); t2022055@njau.edu.cn (H.C.); guanpang@njau.edu.cn (G.P.); wangy@njau.edu.cn (Y.W.); 2 National Engineering and Technology Center for Information Agriculture, MARA Key Laboratory of Crop System Analysis and Decision Making, MOE Engineering Research Center of Smart Agriculture, Jiangsu Key Laboratory for Information Agriculture, Institute of Smart Agriculture, Nanjing Agricultural University, Nanjing 210095, China; zhujie23@njau.edu.cn (J.Z.); yaoxia@njau.edu.cn (X.Y.); 3 Department of Electrical Engineering and Computer Science, University of Michigan, Ann Arbor, MI 48109, USA; peizhang@njau.edu.cn

**Keywords:** RGB imaging, non-destructive measurement, fruit phenotyping, image segmentation

## Abstract

Greenhouse tomato production requires frequent, fruit-level information on maturity, quality, and yield, yet conventional assessment is often destructive, labor-intensive, or dependent on costly sensing hardware. This study presents a lightweight RGB-based framework for non-destructive measurement of greenhouse tomato fruits under foliage occlusion, uneven illumination, and fruit overlap. By improving instance segmentation while constraining computational cost, the framework generated cleaner fruit masks for downstream color analysis, quality inference, and single-fruit weight estimation. Compared with YOLO11n, the proposed model reduced GFLOPs from 10.2 to 8.2 and improved mask precision from 78.2% to 84.6% and mask F1-score from 82.8% to 85.2%, while box recall, mask recall, and IoU showed small trade-offs. Segmentation remained comparatively stable across maturity stages. RGB features from the refined masks were most useful for vitamin C and soluble solids, supporting rapid, indirect quality screening. In parallel, disk-based geometric reconstruction from fruit contours estimated single-fruit weight with MAE values of 1.69 g·fruit^−1^ for large-fruited tomatoes and 1.29 g·fruit^−1^ for cherry tomatoes. The improved masks enhanced single-fruit weight estimation, whereas biochemical quality prediction remained an indirect, trait-dependent estimation that may partly reflect maturity-related color variation rather than direct measurement. Overall, the results demonstrate that improving segmentation fidelity can strengthen downstream trait estimation without increasing sensing complexity, providing a practical RGB-based solution for automated greenhouse tomato phenotyping, while also highlighting the need for standardized imaging and cautious interpretation of biochemical trait predictions.

## 1. Introduction

Greenhouse tomato (*Solanum lycopersicum* L.) production requires timely and precise management to ensure stable yield and consistent fruit quality [1]. Quantitative and repeatable measurement of fruit number, maturity stage, quality attributes, and size-related characteristics is essential for harvest scheduling, grading, and production management [2]. However, these traits are still mainly obtained through manual inspection or destructive sampling, which are labor-intensive, subjective, and difficult to implement for continuous monitoring. The lack of efficient and reliable measurement approaches limits dynamic assessment of fruit development and reduces the potential of data-driven greenhouse management.

RGB imaging has been widely applied in greenhouse tomato monitoring due to its low cost, simple acquisition process, and compatibility with existing production facilities [3]. Most previous studies have focused on fruit detection and counting using object localization methods [3,4,5]. Although effective for estimating fruit number, these approaches provide insufficient information for quantitative fruit measurement, because maturity evaluation, quality assessment, and weight estimation require accurate extraction of fruit color and geometric characteristics. Under greenhouse conditions, foliage occlusion, fruit overlap, illumination variation, and reflective interference frequently introduce background contamination into detected regions, resulting in measurement errors in subsequent feature extraction. This limitation is particularly evident during intermediate ripening stages, when visual differences between fruits and surrounding tissues are relatively weak [6,7]. Tomato fruits generally exhibit smooth surfaces and relatively uniform coloration, and the critical visual information distinguishing fruits from surrounding organs is mainly concentrated near object boundaries [8]. Therefore, reliable boundary delineation is a key prerequisite for reducing segmentation-induced errors and improving the accuracy of image-based fruit measurement.

Instance-level segmentation provides an effective strategy for improving image-based measurement by separating individual fruits from complex backgrounds at pixel resolution while preserving object geometry [6]. Compared with conventional detection approaches, accurate segmentation enables cleaner extraction of fruit regions and more reliable measurement of color and morphological characteristics. Therefore, segmentation methods capable of maintaining contour continuity and regional consistency are essential for robust fruit measurement under variable greenhouse conditions.

Fruit maturity is a critical indicator for harvest scheduling and market grading. In practical production, maturity is commonly classified into three stages: green, breaker, and red, which reduces annotation complexity compared with more detailed grading systems [9,10,11]. In addition to external appearance, internal quality attributes, including vitamin C content, soluble solids, and acidity, determine nutritional and sensory properties and are closely associated with fruit development [12]. Therefore, integrating image-derived characteristics with measured quality traits provides a potential non-destructive approach for fruit quality assessment [7]. Furthermore, fruit weight measurement requires accurate geometric characterization. Although fruit counting provides a basic estimation of production potential, individual fruit size contributes substantially to final yield variation [12]. Geometric parameters derived from accurate fruit contours, such as projected area and size-related descriptors, are closely correlated with fruit weight [4]. Consequently, high-fidelity contour extraction is essential for reliable geometric measurement and weight estimation.

Based on these considerations, this study develops a lightweight RGB-based measurement framework for greenhouse tomato fruits. The objectives are to: (i) develop an instance-level segmentation approach to obtain accurate fruit regions and reduce measurement errors caused by background interference; (ii) establish fruit maturity assessment based on segmented regions across green, breaker, and red stages; and (iii) integrate RGB-derived visual features and geometric characteristics to achieve non-destructive estimation of fruit quality attributes and individual fruit weight. Previous RGB-based studies on tomato phenotyping have often targeted detection, counting, or a single trait. Here, we combine lightweight segmentation with fruit-level phenotyping, quality inference, and weight estimation in a practical low-cost pipeline. Improving the fruit mask at an early stage helps reduce downstream measurement errors without adding new hardware.

## 2. Results

### 2.1. Segmentation Performance Improvement Under Complex Greenhouse Conditions

To improve segmentation reliability under dense occlusion and illumination variation, different lightweight segmentation configurations were evaluated based on the YOLO11n framework. The objective was not only to improve segmentation accuracy but also to preserve contour integrity and reduce feature contamination in downstream phenotyping tasks. YOLO11n was selected as the baseline because it provided a relatively favorable balance between computational efficiency and segmentation performance.

Three structural modifications were subsequently evaluated, including DWConv, C3K2_GSConv, and their combined configuration (NAU). As shown in Table 1, the baseline YOLO11n achieved an mAP@0.5 of 89.1%, an F1 score of 81.7%, and a computational cost of 10.2 GFLOPs. Replacing standard convolution with DWConv alone or introducing C3K2_GSConv individually reduced computational cost, but segmentation performance declined to varying degrees, particularly in mAP@0.5 and F1 score. These results indicate that single lightweight modifications were insufficient to maintain stable contour extraction under complex greenhouse conditions.

In contrast, the combined NAU structure reduced computational cost to 8.2 GFLOPs while improving Mask Precision to 80.9% and slightly increasing the F1 score to 81.9%. The improvement suggests that coordinated lightweight feature extraction and multi-scale feature fusion enhanced contour continuity while suppressing background interference from leaves, stems, and overlapping fruits. As a result, segmentation fidelity was improved without substantially increasing computational burden.

Figure 1 presents training and validation curves of different models. Training loss drops sharply at first and stabilizes after around 50 epochs for most configurations. Compared with the baseline YOLO11n, the YOLO11n-NAU model exhibited faster convergence and lower training loss, indicating improved feature representation efficiency. Validation loss for most modified models stabilized after around 10 epochs, whereas YOLO11n required approximately 25 epochs to reach convergence.

Further optimization using a learning-rate restart strategy produced the YOLO11n-NAU* configuration, which achieved the best overall performance. Box Precision increased to 84.1%, Mask Precision to 84.6%, mAP@0.5 to 89.8%, and F1 score to 84.7%, while GFLOPs remained at 8.2. These results demonstrate that improved segmentation fidelity can be achieved while maintaining lightweight deployment capability, thereby supporting reliable downstream extraction of chromatic and geometric features.

Table S1 compares segmentation performance across different tomato maturity stages. At the immature stage, YOLO11n-NAU achieved the highest precision (90.3%) and mAP@0.5 (94.5%), outperforming all other models. Although recall (86.2%) was slightly lower than that of the baseline model (92.4%), the improved precision indicates stronger suppression of background interference and reduced false segmentation.

The performance improvement was particularly evident during the semi-ripe stage, where subtle chromatic transitions and incomplete color separation commonly increase segmentation uncertainty. YOLO11n-NAU achieved the highest precision (84.1%), recall (84.4%), mAP@0.5 (88.8%), and F1 score (84.25%), demonstrating more stable contour extraction under visually ambiguous conditions.

At the ripe stage, YOLO11n-NAU again showed clear advantages. Precision increased to 80.2%, representing an improvement of 14.9 percentage points relative to the baseline model. Meanwhile, F1 score reached 83.69% and mAP@0.5 reached 87.7%, while maintaining competitive recall performance (87.5%). These results indicate that improved contour continuity effectively reduced segmentation confusion among adjacent mature fruits. YOLO11n-NAU* improved boundary quality and weight prediction, but not all quality traits. Copula augmentation helped only protein and acidity.

Overall, YOLO11n + NAU achieved superior segmentation performance for semi-ripe and ripe fruits while maintaining stable performance for immature fruits. In contrast, models incorporating only DWConv consistently performed worse than the baseline, suggesting that lightweight convolution alone could not maintain sufficient segmentation fidelity under complex greenhouse conditions. Furthermore, as shown in Figure 2, YOLO11n-NAU* demonstrated stronger robustness under occlusion and illumination interference than both YOLO11n and the standard YOLO11n-NAU configuration.

### 2.2. Comparison with Mainstream Segmentation Models

Table 2 compares the proposed framework with several mainstream instance segmentation models in terms of segmentation accuracy and computational efficiency. Unlike conventional benchmark comparisons focusing solely on detection accuracy, this study further emphasized maintaining segmentation fidelity under computational constraints to support reliable downstream phenotyping tasks.

Mask R-CNN, as a representative two-stage segmentation framework, achieved stable recall performance (84.7%) and an mAP@0.5 of 88.4%. However, its computational cost reached 246.1 GFLOPs with 44.1 million parameters, substantially limiting deployment efficiency in greenhouse applications. YOLACT reduced computational burden relative to Mask R-CNN but exhibited comparatively low precision and F1 score, resulting in moderate overall segmentation performance. MobileNet achieved the lowest computational cost (0.7 GFLOPs), but its mAP@0.5 was only 57.1%, indicating insufficient capability for handling occlusion, overlap, and background interference.

Compared with these models, single-stage YOLO-based frameworks achieved a more balanced trade-off between segmentation accuracy and computational efficiency. YOLOv8n and YOLO11n maintained relatively small parameter scales while achieving mAP values above 88%.

Building on this foundation, the proposed YOLO11n + NAU* framework further improved segmentation performance. Precision increased to 84.6%, F1 score to 84.71%, and mAP@0.5 to 89.8%, while computational cost decreased to 8.2 GFLOPs and parameter count decreased to 2.0M. These results indicate that the proposed framework improved contour consistency and segmentation robustness without sacrificing deployment efficiency, thereby providing more reliable fruit masks for downstream phenotyping and estimation tasks.

### 2.3. Fruit Quality Estimation Based on Segmented RGB Features

Figure 3 presents Pearson correlation coefficients between RGB channel features extracted from segmented fruit masks and measured fruit quality traits. Overall, R, G, and B channel values were significantly positively correlated (*p* < 0.05) with fruit firmness, vitamin C content, titratable acidity, and protein content. Among them, the G channel showed relatively strong correlations with firmness (r = 0.63) and vitamin C (r = 0.55), whereas the R channel exhibited the highest correlation with protein content (r = 0.58). In contrast, soluble solids content showed significant negative correlations with RGB features, particularly for the G channel (r = −0.66).

These results indicate that RGB-derived chromatic information effectively reflects physiological and biochemical differences among fruits. The improved segmentation masks reduced background-induced color contamination, allowing RGB features to more consistently represent intrinsic fruit characteristics and thereby improving the reliability of downstream quality estimation.

Model performance was trait-dependent, and no single regression algorithm performed best across all quality indicators (Table 3). Without Copula augmentation, the best test-set performance was obtained for titratable acidity (XGBoost, R^2^ = 0.91), firmness (RF, R^2^ = 0.90), protein (SVM, R^2^ = 0.88), vitamin C (XGBoost, R^2^ = 0.85), and soluble solids (RF, R^2^ = 0.65). With Copula augmentation, the best model changed for some traits: protein and titratable acidity were slightly improved (RF, R^2^ = 0.89 and 0.93, respectively), whereas soluble solids, vitamin C, and firmness decreased (R^2^ = 0.51, 0.83, and 0.84, respectively, vs. 0.65, 0.85, and 0.90 without augmentation). These results indicate that Copula augmentation did not consistently improve prediction on real independent test samples. SHAP analysis was further used to interpret the SVM, RF and XGBoost models (Figures S1–S9). Dependence plots revealed nonlinear responses of individual RGB features, summary plots showed the direction and magnitude of feature contributions, and feature-importance rankings quantified the relative influence of the R, G and B channels on each quality trait.

The results demonstrate that all three models successfully captured nonlinear relationships between RGB features and fruit quality traits, whereas ensemble tree-based models showed stronger capability for identifying stable feature contributions and improving estimation reliability.

### 2.4. Single-Fruit Weight Estimation Based on Segmented Contours

Fruit weight estimation was conducted using convex hull–refined segmentation masks obtained from the proposed framework. To improve geometric consistency, convex hull operations were first applied to reconstruct incomplete or partially occluded fruit contours.

Based on the training dataset, a linear regression relationship between fruit volume (V) and fruit weight (W) was established (Figure S10). Figure S10 presents the performance of the proposed fruit weight estimation approach (R^2^ = 0.91). For large-fruited tomatoes, RMSE and MAE reached 2.86 g·fruit^−1^ and 1.69 g·fruit^−1^, respectively. For cherry tomatoes, RMSE and MAE were 1.68 g·fruit^−1^ and 1.29 g·fruit^−1^, respectively. Compared with YOLO11n masks, YOLO11n-NAU* masks improved firmness prediction (R^2^ = 0.90 vs. 0.88) and single-fruit weight estimation (R^2^ = 0.82 vs. 0.79; RMSE = 21.40 vs. 23.09 g; MAPE = 29.78% vs. 31.08%, Table S2). For vitamin C, soluble solids, acidity and protein, the differences were small and trait-dependent (Table S3).

Overall, the proposed framework achieved stable weight estimation performance for both fruit types. Although absolute estimation errors differed between large-fruited and cherry tomatoes, proportional estimation error was relatively lower for large-fruited tomatoes because of their higher average fruit weight. These results indicate that improved contour continuity and mask completeness contributed directly to more stable geometric reconstruction and fruit weight estimation, further demonstrating the importance of segmentation fidelity for downstream phenotyping tasks.

## 3. Discussion

### 3.1. Segmentation Fidelity as a Key Factor Determining Image-Based Measurement Reliability

In greenhouse environments, segmentation uncertainty directly affects subsequent quantitative measurements by introducing errors into extracted color and geometric features. Accurate instance-level segmentation is therefore a critical prerequisite for reliable image-based assessment of tomato fruit traits. Unlike image classification or bounding-box detection, instance segmentation provides pixel-level fruit delineation, enabling more precise extraction of morphological and chromatic information. This capability is particularly important under greenhouse conditions characterized by foliage occlusion, fruit overlap, reflective surfaces, and illumination variation, where inaccurate boundaries can introduce background contamination and reduce measurement reliability.

Existing segmentation approaches still face limitations under complex greenhouse scenarios. Mask R-CNN can generate pixel-level masks but requires substantial computational resources and may suffer from mask confusion in highly overlapping scenes (>70% overlap), limiting its suitability for real-time measurement applications [13]. YOLOv8 and related one-stage frameworks provide faster inference but often lack sufficient boundary refinement, resulting in incomplete or irregular object contours, especially during breaker-stage transitions where visual differences between fruits and surrounding tissues are limited [14]. UNet-based semantic segmentation methods perform well in relatively simple environments but may experience reduced accuracy when immature fruits resemble stems, leaves, or reflective backgrounds [15].

The proposed YOLO11n-based framework improves measurement reliability by enhancing both contour preservation and feature discrimination. The lightweight convolution strategy strengthens the extraction of edge gradients and subtle color transitions, improving contour continuity and reducing boundary ambiguity among adjacent fruits. This function is particularly important for semi-ripe fruits, where incomplete color transformation increases uncertainty in object delineation. Meanwhile, improved feature representation enhances separation between fruit regions and complex backgrounds, reducing interference caused by leaves, stems, and reflective surfaces. The ablation results further clarify the source of the observed improvement. The structural modification alone mainly reduced computational cost, but it did not consistently improve accuracy: mAP@0.5 decreased from 89.1% to 87.5%, while Mask F1 remained similar (82.8% to 82.6%). A clearer accuracy gain was obtained only when the learning-rate restart strategy was applied to the modified model, increasing mAP@0.5 to 89.8% and Mask F1 to 85.2%. Therefore, the improvement should be attributed to the combined effect of lightweight structural design and optimized training schedule, rather than to the architecture alone.

These improvements explain why enhanced segmentation performance resulted in more reliable downstream measurements. More accurate contours reduced geometric distortion during volume reconstruction, while cleaner fruit regions improved the consistency of RGB feature extraction for quality estimation. Therefore, segmentation quality should not be considered only as an independent recognition metric, but also as an important error-control component in image-based measurement systems. By improving measurement accuracy at the initial image processing stage, the proposed framework provides cumulative benefits for maturity assessment, quality estimation, and weight measurement.

### 3.2. Application Potential of RGB-Based Non-Destructive Fruit Measurement

The proposed framework establishes an integrated image-based measurement approach that combines low-cost RGB sensing, accurate fruit delineation, and quantitative estimation of fruit traits. Unlike conventional manual measurements or destructive sampling methods, the proposed approach enables automated and non-destructive assessment of maturity, quality-related characteristics, and individual fruit weight, providing a practical solution for greenhouse tomato production [16,17].

The selection of RGB imagery as the sole sensing source was based on considerations of measurement accessibility and deployment feasibility. Although hyperspectral and LiDAR-based systems can provide additional spectral or structural information [18], their application in commercial greenhouse environments is often constrained by higher equipment costs, complex calibration requirements, and increased operational maintenance. Tang et al. [19] further indicated that long-term stability and system complexity remain important limitations for multimodal sensing platforms. In contrast, RGB cameras are widely available in greenhouse production systems and can be readily integrated into existing monitoring facilities without substantial modification.

The results demonstrate that reliable measurement does not necessarily require complex sensing systems when the quality of image information extraction is sufficiently improved. By reducing background contamination and preserving fruit boundaries, the proposed framework enabled RGB features to better represent intrinsic fruit characteristics, supporting reliable maturity assessment and quality-related trait estimation. The three-stage maturity classification strategy (green-breaker-red) provides a practical balance between physiological relevance and annotation consistency. Compared with more detailed grading systems that increase labeling complexity [20] and binary classification approaches that simplify fruit development [21], this classification scheme better corresponds to practical harvesting stages.

For quality estimation, RGB-derived features provide indirect measurements of internal fruit properties by capturing visible changes associated with ripening processes. During tomato development, changes in pigmentation and surface appearance are closely related to biochemical transitions, allowing image-derived characteristics to reflect variations in quality attributes [22]. Improved segmentation fidelity further enhances this relationship by reducing the influence of non-fruit pixels during feature extraction [23]. Therefore, the proposed framework improves not only model performance but also the reliability of visual indicators used for quality assessment.

For weight estimation, the proposed method follows a contour–volume–weight pathway. Unlike counting-based approaches that treat fruits as equivalent units [24], contour-based geometric reconstruction uses fruit size and shape information [25]. When the contour is stable, convex-hull filling and disk-based volume calculation are also more stable, which benefits single-fruit weight estimation. Better masks mainly improve geometry-based weight estimation, whereas RGB quality prediction remains indirect and trait-dependent. Occlusion remains a relevant factor. The supplementary examples suggest that filling is helpful under slight or moderate occlusion, but when a large part of the fruit is invisible, the filled contour may include background and overestimate the projected area and volume. Because our dataset contained only a few heavily occluded fruits, this effect could not be quantified. In practice, convex-hull reconstruction should be limited to slight or moderate occlusion; heavily occluded fruits should be excluded or imaged again from another angle. More samples with severe occlusion are needed to evaluate this bias in future work. Better masks mainly improve geometry-based weight estimation; RGB quality prediction remains indirect and trait-dependent.

Altogether, this study establishes an RGB-based measurement framework that improves fruit delineation and supports automated, non-destructive estimation of tomato fruit traits in greenhouse production. However, RGB-based prediction of internal biochemical traits should be regarded as indirect estimation rather than direct measurement, because surface color is influenced by maturity-related pigment changes, peel properties, water status, cultivar, and illumination conditions. As a result, part of the predicted quality signal may reflect ripening-driven color variation rather than true biochemical differences. Likewise, two-dimensional projections cannot fully represent three-dimensional fruit morphology. Nevertheless, improving segmentation fidelity reduces measurement uncertainty at the image-processing stage and improves downstream trait estimation. Future work should combine the proposed framework with standardized imaging or radiometric calibration, larger cultivar- and maturity-balanced datasets, and, where necessary, complementary sensing modalities to improve robustness and biochemical prediction accuracy.

In this study, the dataset included only two tomato cultivars and one greenhouse location, and the number of real samples was limited for some quality traits. RGB measurements may also be affected by illumination, and RGB-based biochemical prediction is indirect. In addition, estimating three-dimensional fruit volume from two-dimensional contours involves simplifying assumptions about fruit shape and occlusion. These factors should be considered when applying the framework to new cultivars, environments, or imaging conditions.

## 4. Materials and Methods

### 4.1. Image Collection

Tomato image data were collected from March 2024 to December 2025 in a greenhouse at the Baima Experimental Station of Nanjing Agricultural University, Nanjing, China. Images were captured via a Canon EOS 6D Mark II camera (Canon Inc., Japan, Tokyo), covering two tomato cultivars: ‘Provence’ (large-fruited) and ‘Zheyinfan 2’ (cherry tomato). The dataset covers a wide range of conditions, including sunny and cloudy weather, morning and late-afternoon illumination, varying levels of fruit overlap, leaf and stem occlusion, different background environments, and multiple maturity stages. Both single-factor scenarios and complex combinations of multiple factors were represented. A total of 311 images were collected in the greenhouse, each with a resolution of 4160 × 4160 pixels. In addition, 841 images were obtained from public datasets (https://www.agridata.cn/data.html#/datadetail?id=289638) (accessed on 13 January 2022) [26]. After data cleaning and quality control, all images were retained in JPG format for subsequent analysis.

### 4.2. Tomato Fruit Maturity Grading

Tomato maturity was classified into six color-based grades according to Chinese industry standard GH/T 1193-2021: green, mature green, breaker, early red, mid-red, and late red. For practical harvesting and analysis, these six grades were collapsed into three stages: green and mature green were grouped as immature; breaker and early red were grouped as semi-ripe; and mid-red and late red were grouped as ripe. Fruits with less than 10% red coloration were defined as immature; these fruits had reached full size with a glossy surface and remained predominantly green or slightly yellow. Fruits with 10% to less than 80% red coloration were defined as semi-ripe, typically showing distinct red-yellow patches or stripes; fruits at this stage are suitable for harvest for transportation or storage. Fruits with 80% or more red coloration were defined as ripe, with an almost entirely red surface and suitability for direct marketing. Under this rule, fruits at exactly 10% red were assigned to the semi-ripe stage, and fruits at exactly 80% red were assigned to the ripe stage. The specific classification criteria are summarized in Figure S11.

### 4.3. Image Preprocessing and Dataset Construction

Original images were annotated using the ISAT-SAM interactive semi-automatic segmentation tool. Two datasets were constructed for different purposes. Dataset A was annotated by maturity stage, including immature, semi-ripe, and ripe fruits, and was used for maturity-stage stratified evaluation where reported. Dataset B treated all tomato fruits as a single foreground category regardless of maturity stage, and was used for fruit detection/instance segmentation and downstream single-fruit phenotyping and weight estimation. All annotation files were exported in TXT format.

The public dataset was divided 7:3 for training and validation. Captured images formed the test set, with data augmentation applied to enhance robustness. Following the strategy of [27], polygon vertices were treated as spatial keypoints and transformed synchronously with corresponding images using the Albumentations library.

Two categories of augmentation were employed. Spatial augmentations included horizontal flipping, vertical flipping, random rotation, shift-scale-rotate transformation, and padding adjustment. Photometric augmentations included Gaussian blur, noise injection, brightness and contrast adjustment, hue-saturation-value transformation, RGB channel shift, CLAHE enhancement, gamma correction, image compression, grayscale conversion, and random region dropout. For each image, two to three augmentation operations were randomly combined to simulate illumination variation, occlusion, and background interference commonly encountered in greenhouse environments. Example augmentation results are shown in Figure S12. After augmentation, the training set contained 1514 images, the validation set contained 84 images, and the test set contained 311 self-collected field images. Detailed image and annotation statistics are provided in Table S4.

### 4.4. Model Training and Evaluation Metrics

The model was developed in Python (3.12.10) with PyTorch (2.9.0+cu130). Hardware is listed in Table S5. Images were resized to 640 × 640, trained for 400 epochs (batch size 32) using Adam optimizer and a one-cycle learning rate. Automatic mixed precision training was adopted to save GPU memory and boost efficiency.

Non-maximum suppression with a threshold of 0.5 eliminated redundant bounding boxes. We adopted box precision/recall, mask precision/recall, mAP@0.5, mIoU, F1-score, GFLOPs and parameter count to evaluate segmentation performance and computational efficiency.

### 4.5. Determination of Tomato Fruit Quality Indicators

Vitamin C was measured via phosphomolybdate colorimetry with a commercial kit, and fruit weight was recorded using an electronic balance. Fruit firmness was measured according to Chapman et al. [28] using a GY-4 digital fruit penetrometer (Zhejiang TOP Cloud-Agri Technology Co., Ltd., Hangzhou, China) equipped with a 3 mm probe. Four measurements were taken at the equatorial region of each fruit, and the average value was recorded. Soluble solids content was measured using a handheld refractometer (SW-35T). Titratable acidity was determined using a portable acidity meter (ATAGO PAL-Easy-ACID3, Aituo, Tokyo, Japan), and protein content was quantified using the Coomassie Brilliant Blue colorimetric method.

A total of 411 real tomato fruits were used for quality modeling. The trait-specific sample sizes were 411 for vitamin C, firmness, titratable acidity and protein content, and 247 for soluble solids content, according to the fruits with valid measurements for each trait. The 411 real fruits were distributed as follows: cherry tomato, 282 fruits (47 immature, 115 semi-ripe, and 120 ripe); large-fruited tomato, 129 fruits (34 immature, 54 semi-ripe, and 41 ripe). To alleviate the limitation of small sample size while pre-serving the statistical structure of the real data, the real samples were first divided into training and test sets at a ratio of 7:3. Copula functions were then fitted using only the training real samples to generate 1343 synthetic training samples, while maintaining marginal distributions and inter-trait correlations. No synthetic samples were included in the test set, and all reported quality-prediction results were evaluated on real independent test fruits only. Model performance was further compared with and without Copula augmentation to evaluate the effect of synthetic-data training on real test fruits (Table S6). For single-fruit weight estimation, 247 fruit images acquired at approximately 50 cm from the fruit were paired with the corresponding measured fruit weights. These image–weight pairs were divided equally into training and test sets, and model performance was evaluated on the independent test set. The sample size and weight distribution for large-fruited and cherry tomatoes are summarized in Table S2. Features from YOLO11n and YOLO11n-NAU* masks were compared using the same downstream protocol. Boundary quality was evaluated using Boundary IoU and boundary F-score in Table S3 (Figure S15).

### 4.6. High-Fidelity Lightweight Segmentation Framework

In greenhouse environments, fruit overlap, occlusion, illumination variation, and reflective interference frequently distort fruit contours and introduce background information into segmented regions, thereby affecting subsequent extraction of chromatic and geometric features. A lightweight YOLO11n-seg-based instance segmentation framework is developed to enhance contour integrity, mitigate feature contamination, and maintain computational efficiency.

Two modifications are made. First, standard convolutions in the Backbone and Neck are replaced with depthwise separable convolutions to cut computational redundancy while retaining spatial information. Second, the GSConv module is embedded into C3K2 to strengthen multi-scale feature fusion and feature consistency under dense occlusion (Figure S14). The overall framework is shown in Figure 4.

### 4.7. Boundary-Preserving Lightweight Convolution Block

The original YOLO11n-seg backbone adopts a sequential structure of “3 × 3 standard convolution → batch normalization → SiLU activation,” which simultaneously performs spatial feature extraction and channel fusion. However, coupling spatial aggregation and channel transformation within a single operation introduces cross-channel redundancy and increases computational cost.

To address this issue, standard convolutions in the Backbone and Neck are replaced with depthwise separable convolution (DSC), as shown in Figure 5. Depthwise convolution extracts spatial features per channel, while 1 × 1 pointwise convolution fuses cross-channel information. This design retains contour details and suppresses background interference under dense occlusion.

Compared with conventional convolution, DSC substantially reduces parameter count and computational burden:

(1) $$P_{c}=k_{w}\times k_{h}\times c\times a$$

(2) $$P_{dc}=k_{w}\times k_{h}\times c+ca$$

where ‘$P_{c}$’ means the number of parameters in a conventional convolutional layer, ‘a’ means the number of filter kernels, ‘$P_{dc}$’ represents the number of parameters in the depthwise separable convolution layer.

When $k_{w}$ ≥ 2, $k_{h}$ ≥ 2, c ≥ 2 (with all values greater than 0), the number of parameters in conventional convolution ($P_{c}$) is greater than that in depthwise separable convolution ($P_{dc}$). This indicates that, compared with standard convolution, depthwise separable convolution requires fewer parameters and incurs lower computational cost.

### 4.8. C3K2_GSConv Block

To further improve computational efficiency and multi-scale feature consistency, the traditional C3K2 module in YOLO11n was enhanced by integrating the lightweight GSConv module. Specifically, standard convolution layers within the Bottleneck structure were replaced with GSConv. The structure of the improved C3K2_GSConv module is shown in Figure S13.

GSConv combines grouped convolution and spatial convolution to reduce parameter redundancy while maintaining effective feature extraction. As illustrated in Figure 6, standard convolution is first applied for preliminary feature extraction and channel reduction. Subsequently, depthwise convolution independently processes each channel to strengthen spatial feature representation with low computational cost. Outputs of the two branches are concatenated and shuffled channel-wise to facilitate cross-channel information exchange. This structure boosts fusion efficiency and contour continuity amid fruit overlap and subtle color variations in greenhouses.

### 4.9. Estimation of Single-Fruit Weight

Tomato images were first processed using the proposed segmentation framework to obtain instance masks. A convex hull operation was then applied to each segmented mask to reconstruct incomplete or partially occluded fruit regions and generate geometrically consistent fruit contours.

Based on the refined mask, the maximum fruit diameter was identified. Fruit volume was subsequently estimated using the method of solids of revolution, also referred to as the disk-slicing method (Figure 7). This approach approximates the fruit as a rotationally symmetric convex body and discretizes the structure into a series of thin cylindrical elements perpendicular to the principal axis.

By integrating the volumes of these infinitesimal disks, three-dimensional fruit volume can be estimated from two-dimensional projected contours while maintaining geometric consistency. Because volume estimation depends directly on contour integrity, accurate instance segmentation is essential for reliable weight estimation.

The principal axis unit vector of the convex hull point set H was defined as ν, and its orthogonal direction was expressed as ν⊥ = [−vy, vx]. The point set H was projected onto ν⊥ to obtain the interval [t_min_, t_max_], which was uniformly sampled with a step size of Δt. At each sampling position t_i_, a scanning line parallel to ν was constructed. The intersection set between the scanning line and the convex hull boundary was defined as: $I_{i}=L\left(t_{i}\right)\cap \partial H$.

The chord length ‘$l_{i}$’ is determined by the maximum distance between the two farthest intersection points in the set ‘I_i_’, as expressed in Equation (3):

(3) $$l_{i}=\underset{p,q\in I_{i}}{max}{∥p-q∥}_{2}$$

The corresponding elemental volume is approximated using the disk method, as expressed in Equation (4):

(4) $$\Delta V_{i}=\pi (\frac{l_{i}}{2}{)}^{2}\Delta t$$

The total fruit volume is then estimated using a Riemann summation over all slices, as expressed in Equations (5) and (6):

(5) $$V=\sum_{i=1}^{N}\pi \frac{l_{i}^{2}}{4}△t$$

(6) $$N=\frac{t_{max}-t_{min}}{△t}$$

If the pixel-to-millimetre scale at working distance *d* is denoted by *s*(*d*), the physical volume is Equation (7):

(7) $$V={s(d)}^{3}\times V_{px}$$

All fruit-weight-estimation images were acquired at a fixed camera–fruit distance of approximately 50 cm. Therefore, the pixel-to-millimetre scale factor *s*(*d*) was treated as constant for this dataset. The explicit physical-volume conversion defined by *s*(*d*) is shown for completeness, but no independent metric calibration was performed; under the fixed imaging geometry, the constant scale effect was absorbed into the empirical regression coefficient between measured fruit weight and mask-derived pixel volume.

The single-fruit weight was then estimated by the fitted linear model Equation (8):

(8) $$W=a\times V+b=a\times {s(d)}^{3}\times V_{px}+b$$

In this study, all fruit-weight-estimation images were acquired at a fixed camera–fruit distance of approximately 50 cm; therefore, *s*(*d*)^3^ was constant and was absorbed into the empirical regression coefficient, giving the equivalent fixed-distance form $W=7.698\times 10^{-8}\times V+5.723$.

### 4.10. Data Analysis and Processing

OriginPro (OriginLab, Northampton, MA, USA) was used for visualization and preliminary statistical analysis. Statistical analysis and weight estimation were performed in R software (version 4.2.2). Scatterplot matrices were generated using the pairs() function, and pairwise correlation coefficients were visualized using a customized panel.cor function. This study used support vector machine (SVM), random forest (RF), XGBoost, and conventional regression models for establishing relationships between RGB-derived features and fruit quality attributes. Model performance was evaluated using the coefficient of determination (R^2^), root mean square error (RMSE), and mean absolute error (MAE).

## 5. Conclusions

This study developed an RGB-only fruit measurement framework to reduce the reliance of greenhouse tomato phenotyping on destructive sampling and high-cost sensing. Rather than pursuing gains on a single benchmark, the framework was designed to improve fruit-level mask fidelity under occlusion, uneven illumination, and fruit overlap while retaining a lightweight computational profile for practical deployment. The optimized network reduced GFLOPs from 10.2 to 8.2 relative to YOLO11n and yielded higher mask precision and mask F1, whereas recall and IoU showed minor decreases or remained similar, suggesting a trade-off between precision and computational efficiency. More importantly, cleaner instance masks provided a better basis for downstream estimation: they improved single-fruit weight prediction and slightly improved firmness prediction, whereas biochemical quality prediction remained trait-dependent and did not consistently benefit from mask refinement or Copula augmentation. In addition, disk-based reconstruction from fruit contours achieved single-fruit weight estimation, with MAE values of 1.69 g for large-fruited tomatoes and 1.29 g for cherry tomatoes. These results suggest that segmentation fidelity can act as an upstream constraint on RGB-based trait estimation. The framework improves weight estimation and provides indirect, trait-dependent quality screening. The proposed framework is therefore best positioned as a low-cost, deployable front end for automated tomato fruit measurement, with further gains expected from standardized imaging, larger cultivar- and site-specific datasets, and explicit treatment of occlusion and the indirect relationship between RGB signals and internal biochemical traits.

## Figures and Tables

**Figure 1:**
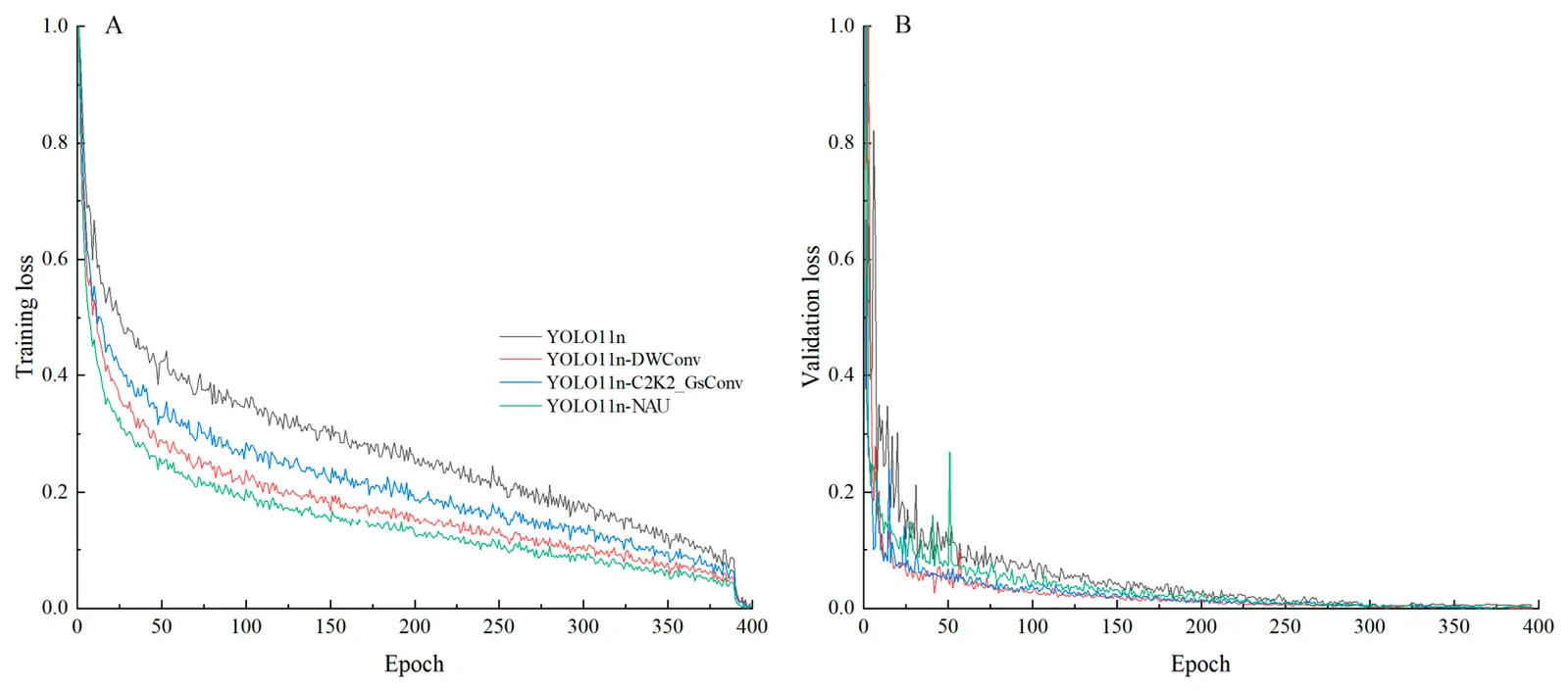
Performance comparison of YOLO11n segmentation models with different improvement modules in various maturity stages of tomatoes. (**A**) The training loss of four different models; (**B**) The validation loss of four different models.

**Figure 2:**
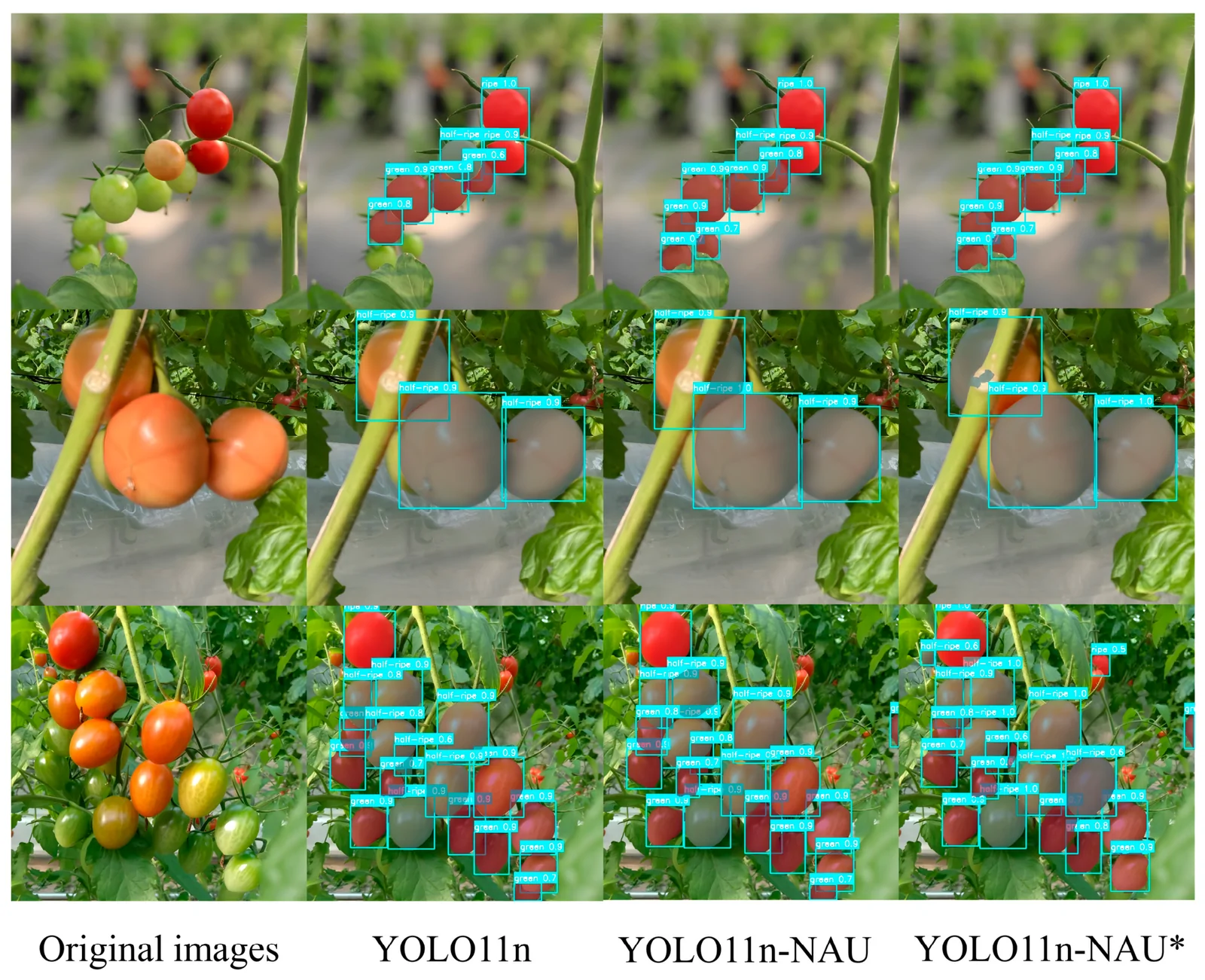
Example segmentation outputs of YOLO11n and the improved models across immature, semi-ripe, and ripe tomato fruits.

**Figure 3:**
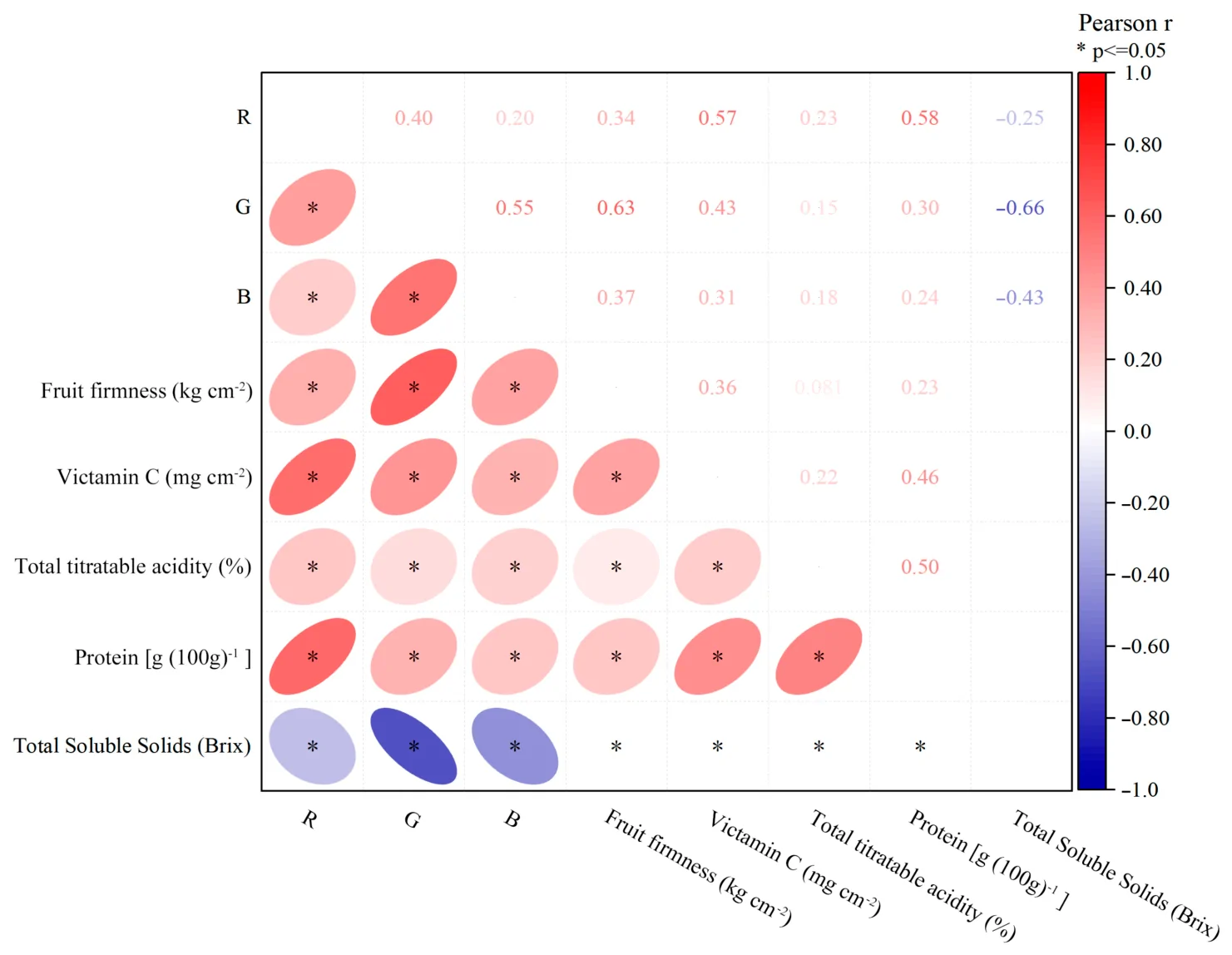
Correlation of segmented fruit RGB images for fruit quality and other indicators.

**Figure 4:**
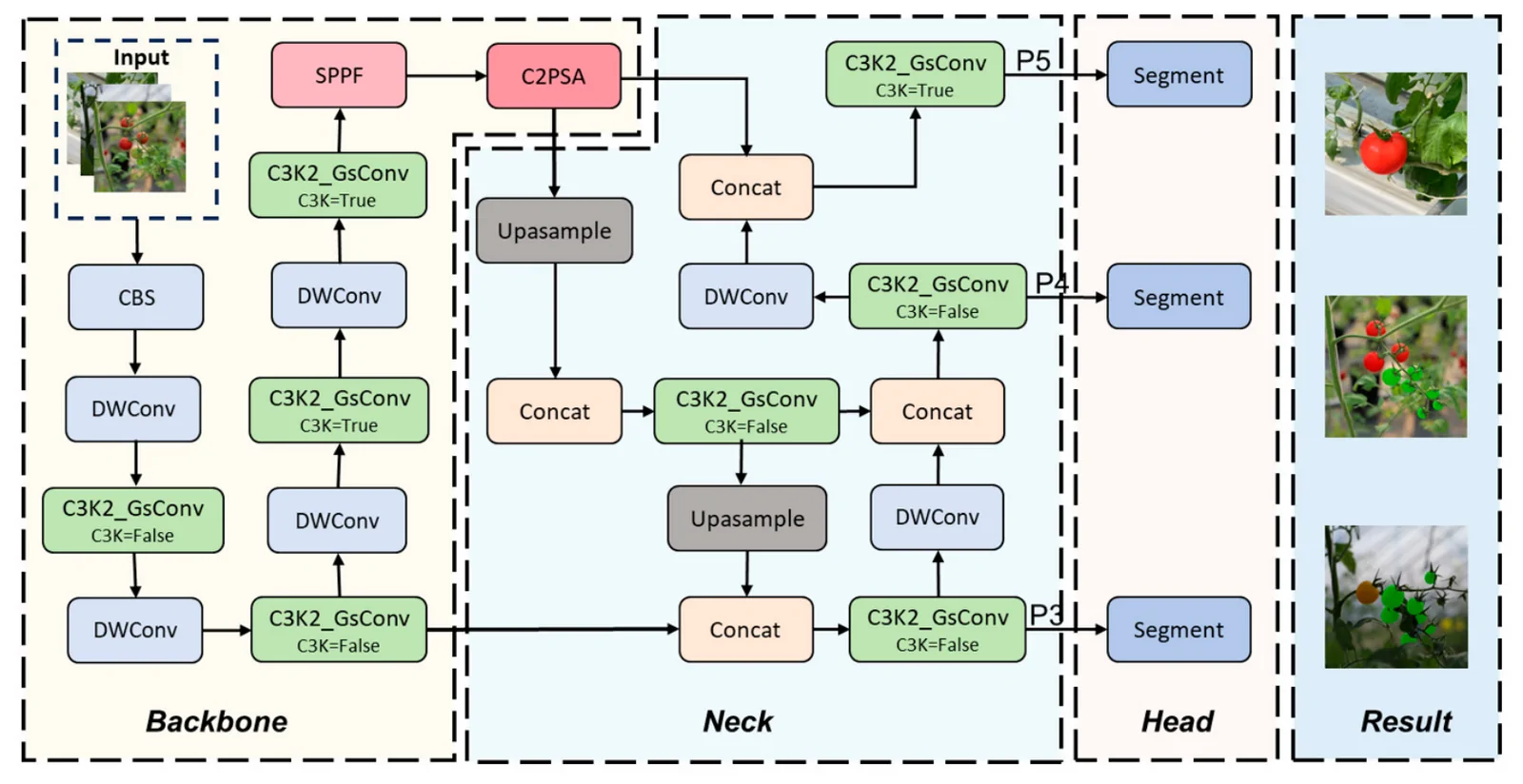
The improved YOLO11n-NAU model structure.

**Figure 5:**
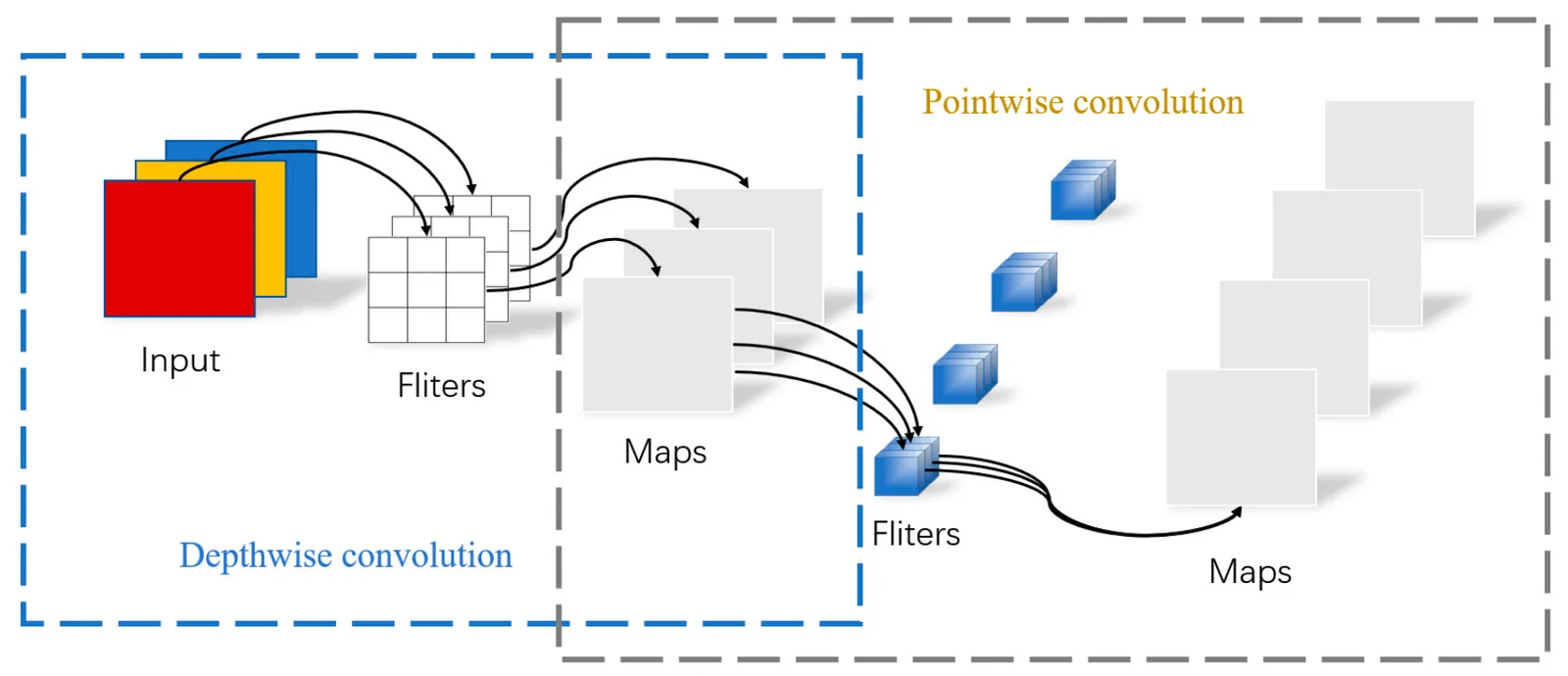
Structure diagram of the depthwise separable convolution module.

**Figure 6:**
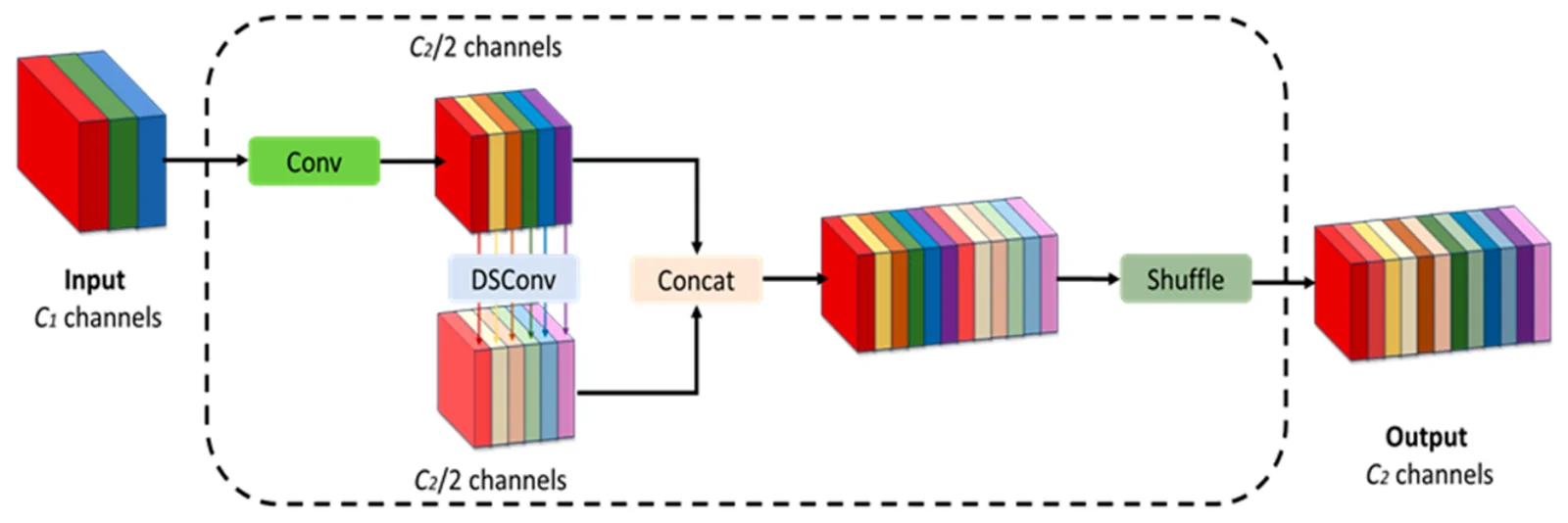
Grouped spatial convolution module structure diagram.

**Figure 7:**
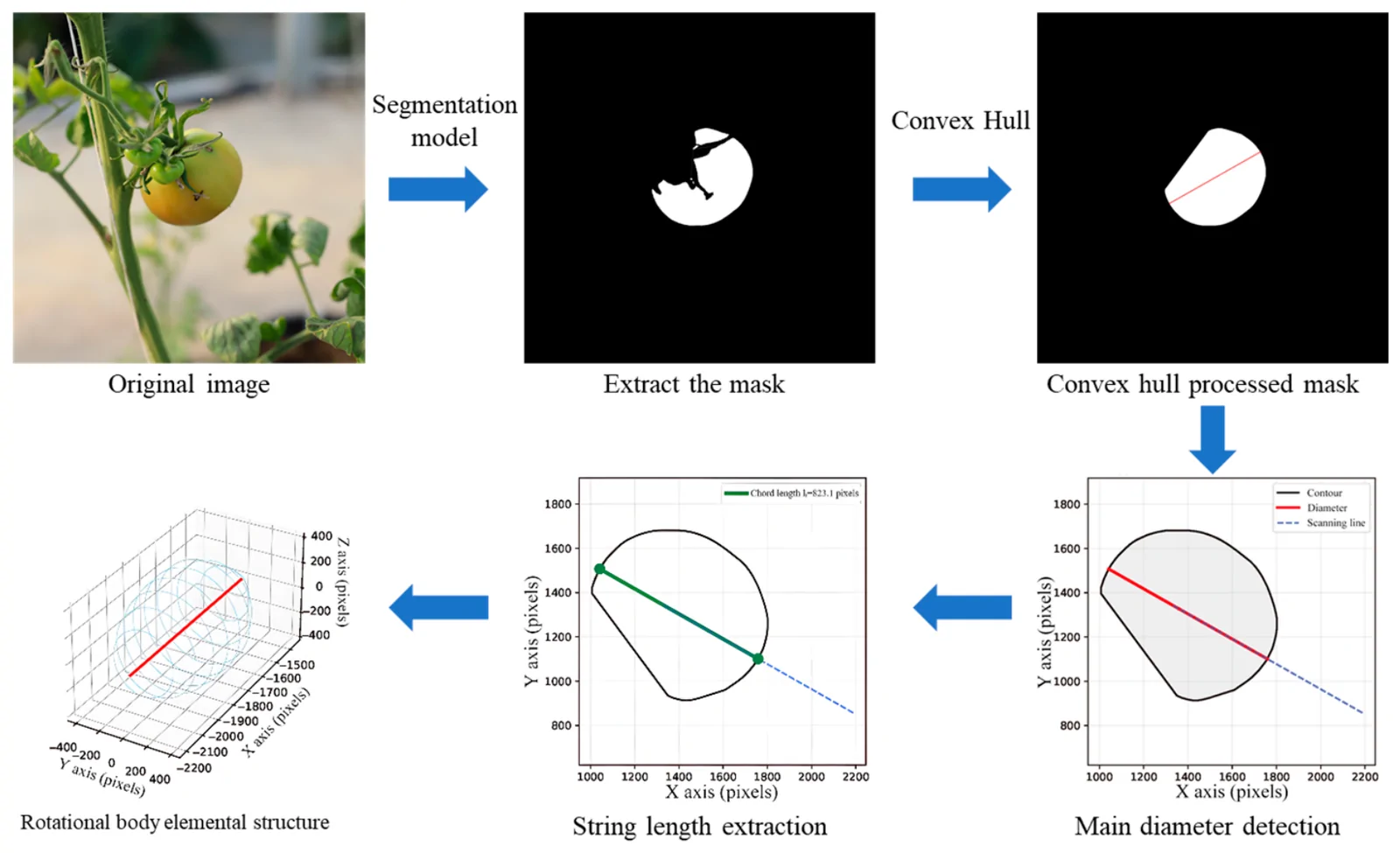
Flowchart for calculating the weight of a single tomato fruit.

**Table 1:** The comprehensive performance of the segmentation model after adding different modules based on YOLO11n.

Model	Box Precision (%)	Box Recall (%)	Mask Precision (%)	Mask Recall (%)	mAP@0.5 (%)	IoU (%)	F1 Score (%)	GFLOPs
YOLO11n	77.8	87.9	78.2	87.9	89.1	92.0	81.7	10.2
YOLO11n + DWConv	74.5	85.8	74.8	86.4	85.6	91.2	78.7	8.6
YOLO11n + C3K2_GsConv	78.4	86.1	78.8	86.4	87.6	91.3	81.5	9.8
YOLO11n + DWConv + C3K2GsConv(NAU)	80.7	84.2	80.9	84.4	87.5	91.2	81.9	8.2
YOLO11n + NAU*	84.1	85.4	84.6	85.9	89.8	90.7	84.7	8.2

Note: YOLO11n + NAU* represents the segmentation results obtained by using the learning rate restart strategy.

**Table 2:** A comparison of the segmentation model in this study with mainstream segmentation models.

Segment Model	Precision (%)	Recall (%)	F1 Score (%)	mAP@0.5	GFLOPs	Parameter Count
Mask R-CNN	73.5	84.7	78.70	88.4	246.1	44.1
YOLACT	55.0	64.4	59.33	84.8	82.7	50.1
MobileNet	82.0	73.1	77.30	57.1	0.7	1.8
YOLOv8n	78.0	87.4	80.84	88.0	12.0	3.3
YOLO11n	78.2	87.9	81.74	89.1	10.2	2.8
YOLO11n + NAU	80.9	84.4	81.93	87.5	8.2	2.0
YOLO11n + NAU*	84.6	85.9	84.71	89.8	8.2	2.0

Note: Results were obtained under the same dataset split, input size and evaluation protocol unless otherwise noted; values taken from previous studies are marked accordingly.

**Table 3:** Quality prediction with and without Copula augmentation.

Trait	Setting	Best Model	RMSE	MAE	R^2^
Firmness	Copula	RF	3.33	1.88	0.84
Firmness	No Copula	RF	2.58	1.38	0.90
Protein	Copula	RF	2.66	1.26	0.89
Protein	No Copula	SVM	2.83	1.37	0.88
Soluble solids/sweetness	Copula	Linear regression	1.01	0.75	0.51
Soluble solids/sweetness	No Copula	RF	0.86	0.63	0.65
Titratable acidity	Copula	RF	2.07	1.21	0.93
Titratable acidity	No Copula	XGBoost	2.35	1.39	0.91
Vitamin C	Copula	SVM	2.91	1.79	0.83
Vitamin C	No Copula	XGBoost	2.72	1.60	0.85

## Data Availability

The datasets and code supporting the findings of this study are available from the corresponding author upon reasonable request. In addition, the relevant code for this study will be hosted in a public GitHub repository (GitHub Desktop).

## Supplementary Materials

The following supporting information can be downloaded at https://www.mdpi.com/article/10.3390/plants15192952/s1, Figure S1 SHAP dependence based on support vector machine. Figure S2 Feature influence based on support vector machine using SHAP. Figure S3 Feature importance based on support vector machine using SHAP. Figure S4 SHAP dependence based on random forest. Figure S5 Feature influence based on random forest using SHAP. Figure S6 Feature importance based on random forest using SHAP. Figure S7 SHAP dependence based on XGBoost. Figure S8 Feature influence based on XGBoost using SHAP. Figure S9 Feature importance based on XGBoost using SHAP. Figure S10 The prediction results of the RGB images of segmented fruits for the yield of individual fruits. Figure S11 Tomato fruit samples at different stages of maturity (a represents Immature stage, the fruit has 0% to 10% red coloration, and is mostly green or shows a slight yellow tint; b represents: semi-ripe stage, The fruit surface is 10% to 80% red in color, with distinct red-yellow spots or stripes; c represents unripe stage, The fruits have 80% to 100% red color, and are basically completely red). Figure S12 Different effects after image enhancement ((a) Original image (b) Random flipping (c) Gaussian noise (d) Random brightness (e) Histogram equalization (f) Random area deletion (g) Shift, scale, rotation (h) Enhanced effect image). Figure S13 The structure diagram of the improved C3K2_GSConv module. Figure S14 Comparison of mask details of different models. Figure S15 Residual distribution of single fruit weight estimation by mask source. Table S1 The segmentation performance across different tomato maturity stages. Table S2 Weight distribution of the training dataset used for single-fruit weight estimation. Table S3 Downstream quality prediction and boundary evaluation using YOLO11n and YOLO11n-NAU masks*. Table S4 Real sample size by cultivar and maturity stage. Table S5 Image processing configuration parameter information. Table S6 Tomato image target information.
