# Supplementary material for: RGB-Based Non-Destructive Measurement of Greenhouse Tomato Fruit Traits Using High-Fidelity Segmentation
Source: Plants (Basel). 2026 Sep 28;15(19):2952. doi: 10.3390/plants15192952 (PMC13666750; doi:10.3390/plants15192952)
Supplement: Supplementary file 1 [file plants-15-02952-s001.zip › plants-4544004-supplementary.pdf]

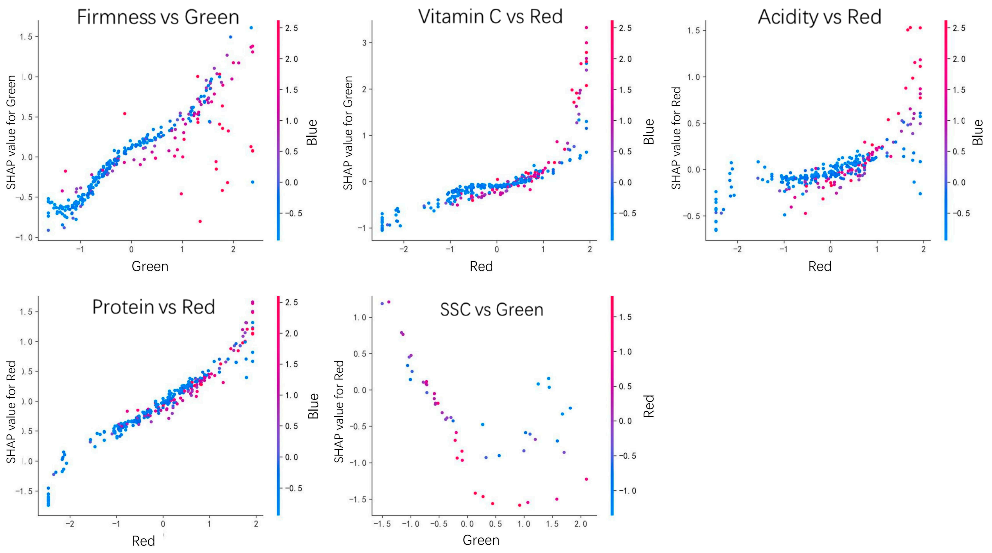

Figure S1 SHAP dependence based on support vector machine

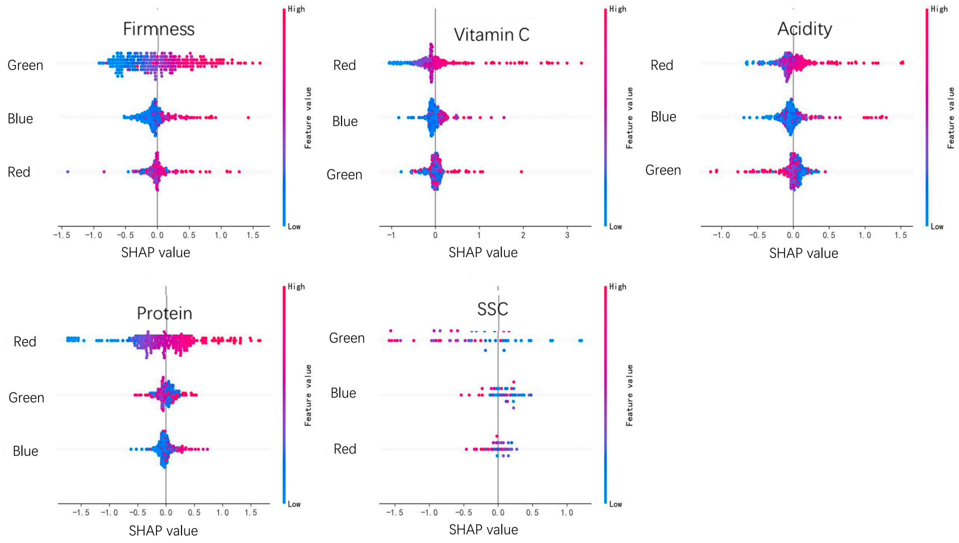

Figure S2 Feature influence based on support vector machine using SHAP

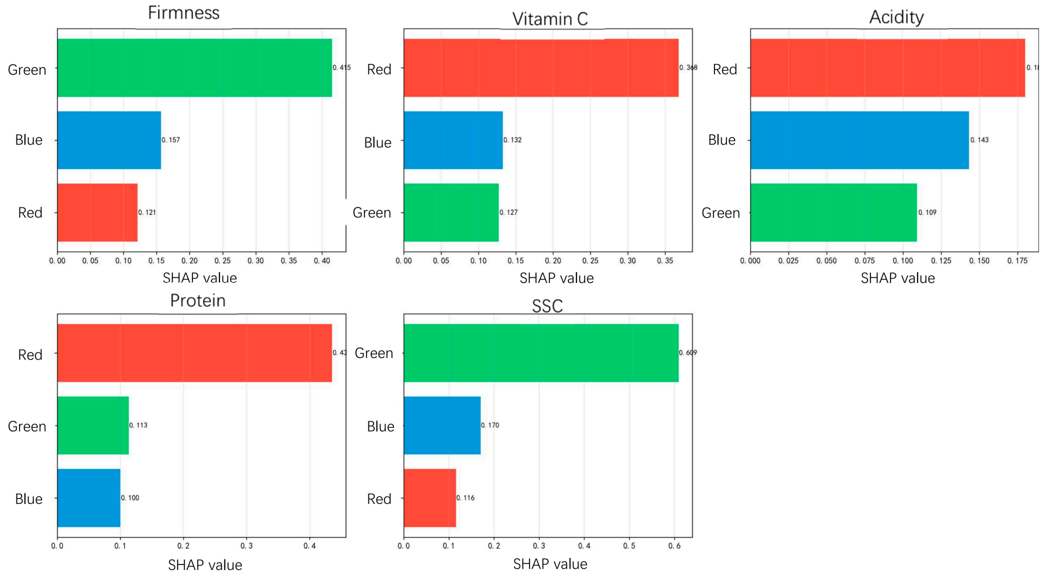

Figure S3 Feature importance based on support vector machine using SHAP

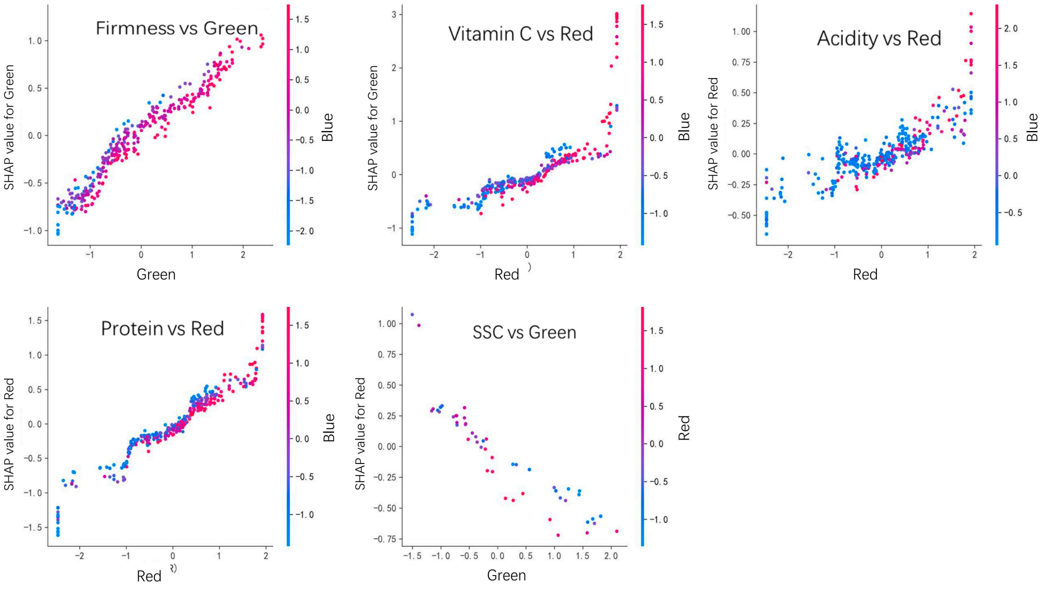

Figure S4 SHAP dependence based on random forest

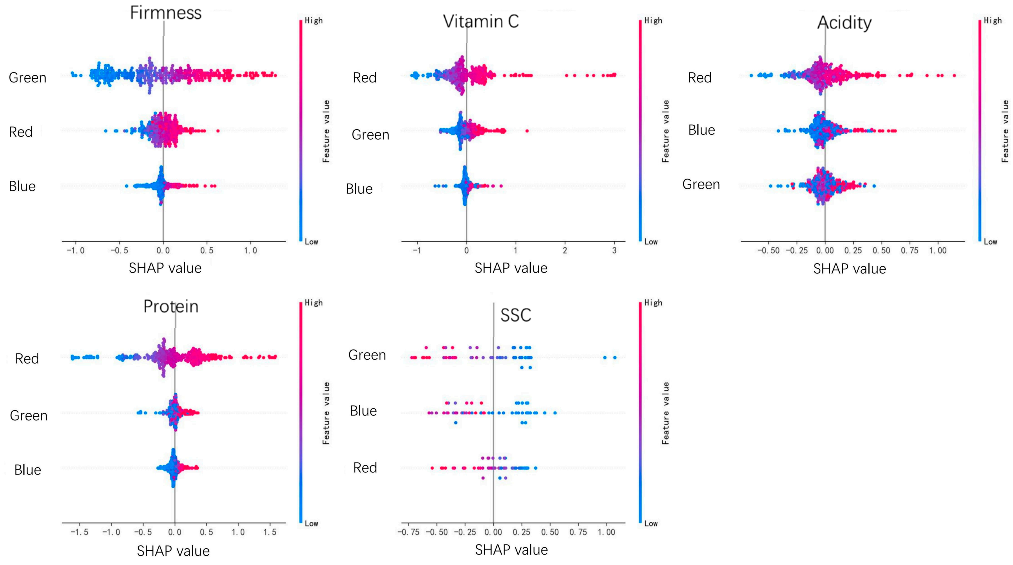

Figure S5 Feature influence based on random forest using SHAP

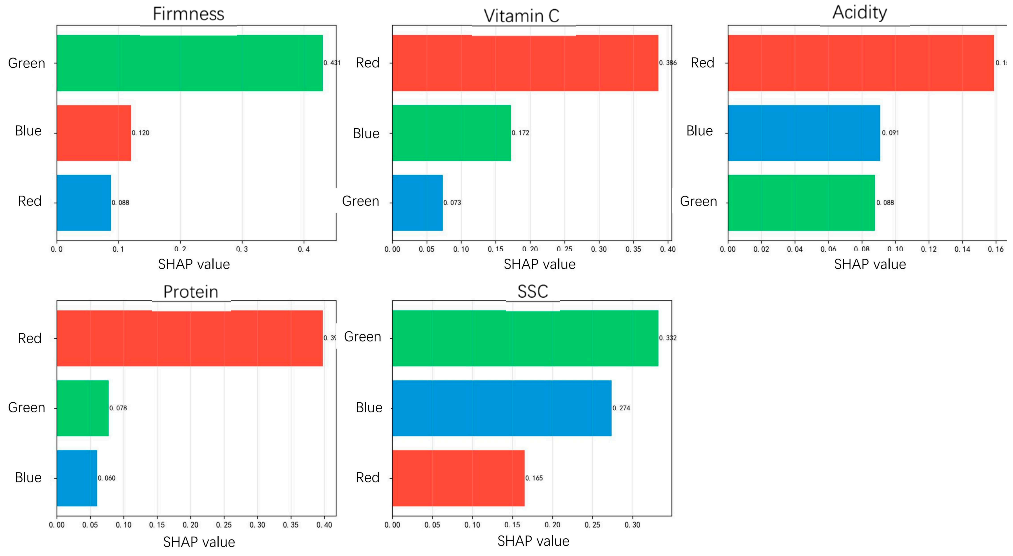

Figure S6 Feature importance based on random forest using SHAP

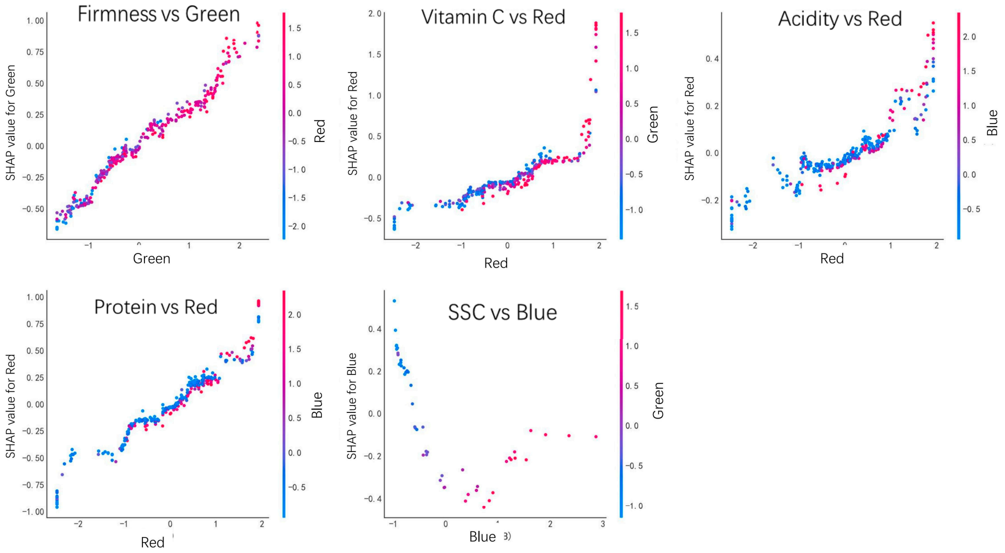

Figure S7 SHAP dependence based on XGBoost

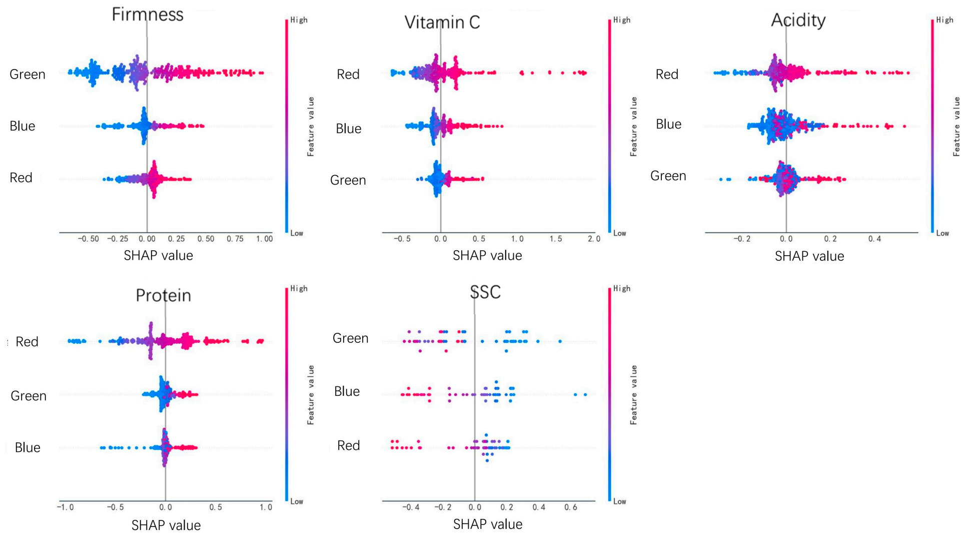

Figure S8 Feature influence based on XGBoost using SHAP

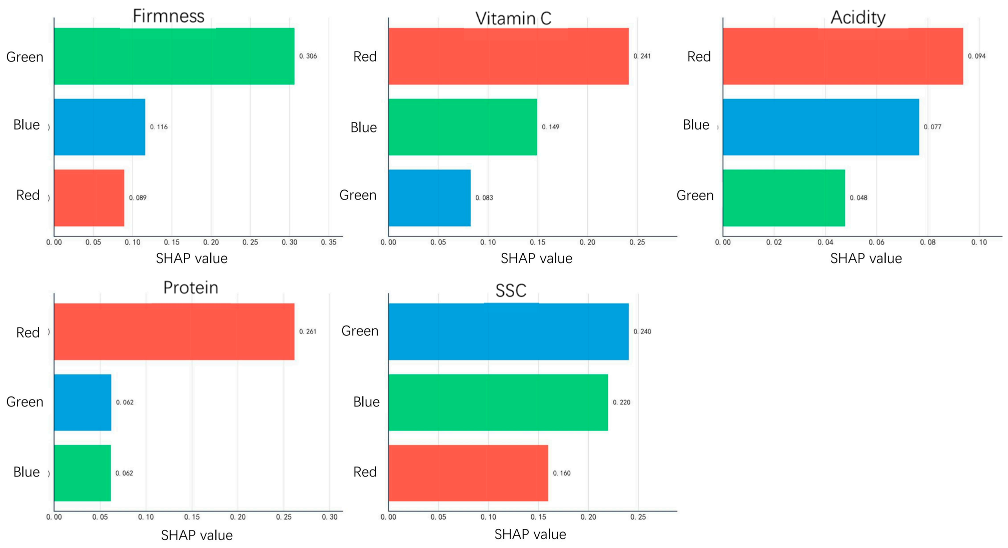

Figure S9 Feature importance based on XGBoost using SHAP

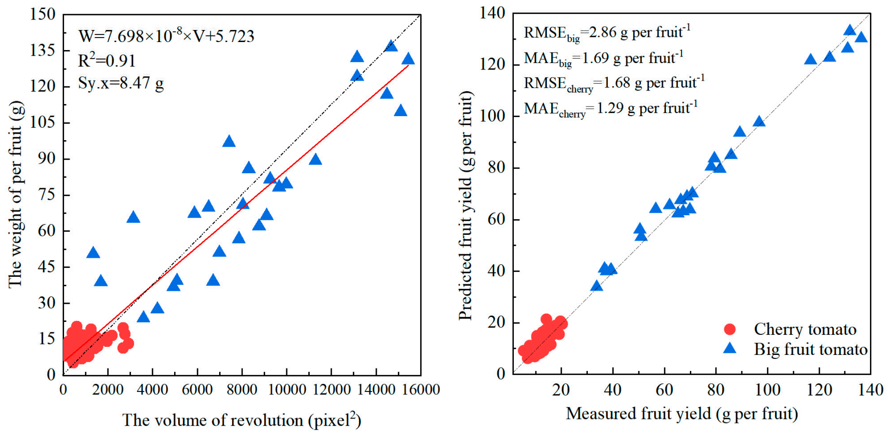

Figure S10 The prediction results of the RGB images of segmented fruits for the yield of individual fruits

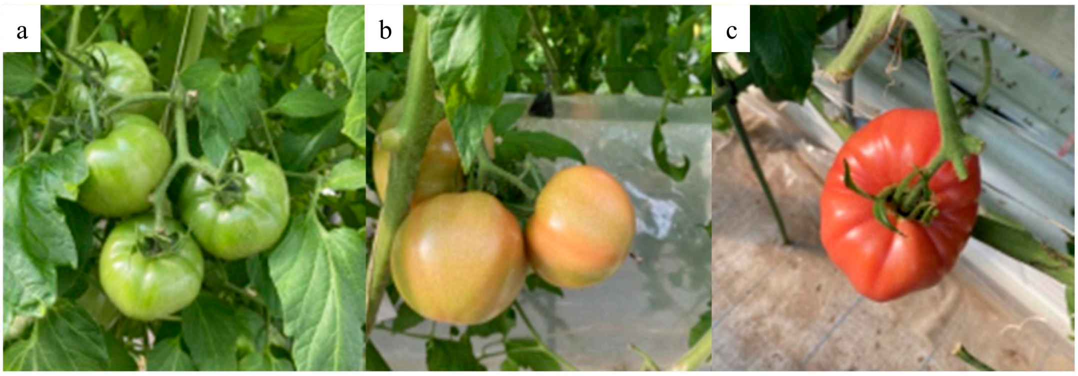

Figure S11 Tomato fruit samples at different stages of maturity (a represents Immature stage, the fruit has 0% to 10% red coloration, and is mostly green or shows a slight yellow tint; b represents: semi-ripe stage, The fruit surface is 10% to 80% red in color, with distinct red-yellow spots or stripes; c represents unripe stage, The fruits have 80% to 100% red color, and are basically completely red)

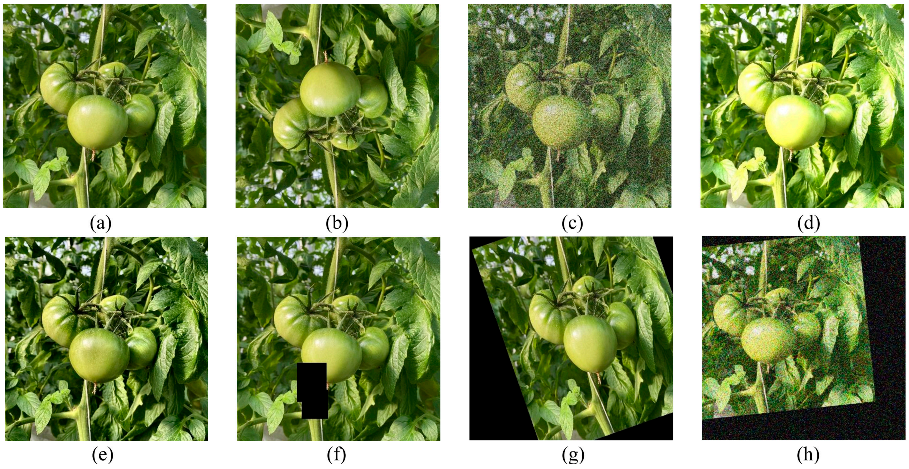

Figure S12 Different effects after image enhancement ((a) Original image (b) Random flipping (c) Gaussian noise (d) Random brightness (e) Histogram equalization (f) Random area deletion (g) Shift, scale, rotation (h) Enhanced effect image)

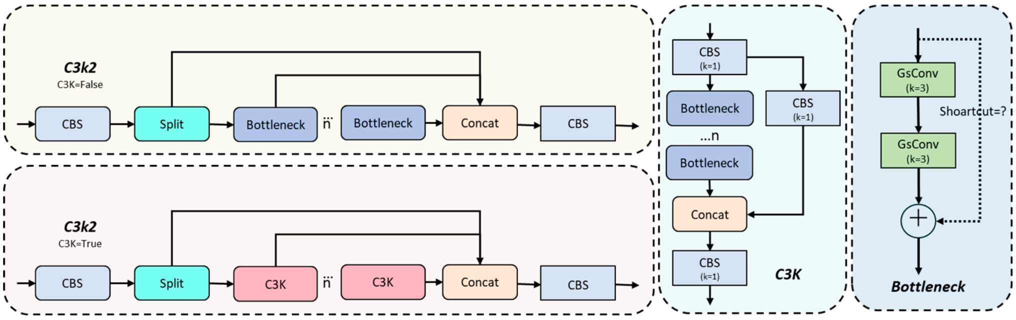

Figure S13 The structure diagram of the improved C3K2_GSConv module

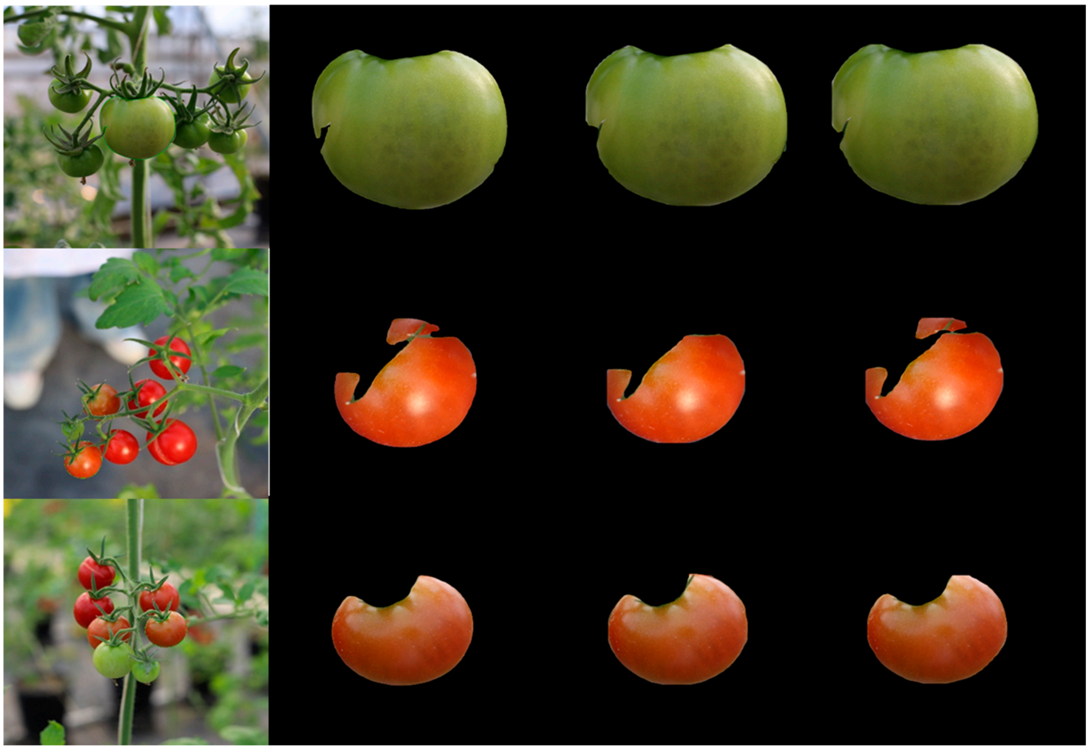

Figure S14 Comparison of mask details of different models

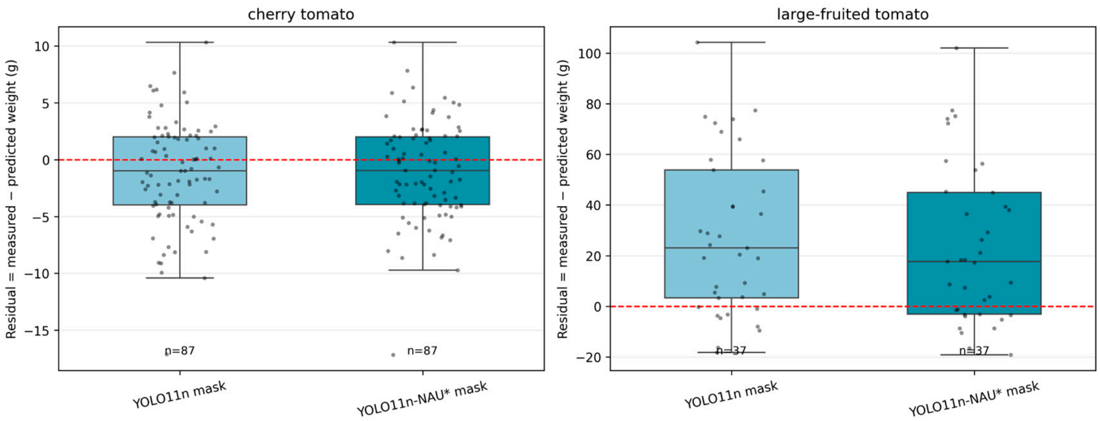

Figure S15 Residual distribution of single fruit weight estimation by mask source

Table S1 The segmentation performance across different tomato maturity stages

Category	Model	Precision (%)	Recall (%)	mAP@0.5	F1 Score (%)
Green	YOLO11n	85.9	92.4	94.2	89.03
	YOLO11n+DWConv	84.3	90.3	92.6	87.20
	YOLO11n+ C3K2_GsConv	87.4	89.1	93.8	88.24
	YOLO11n+NAU	90.3	86.2	94.5	88.20
	YOLO11n+NAU *	89.5	85.9	92.9	87.66
Half-ripe	YOLO11n	83.5	75.3	86.4	79.19
	YOLO11n+DWConv	80.0	73.4	82.7	76.56
	YOLO11n+ C3K2_GsConv	81.1	75.9	84.3	78.41
	YOLO11n+NAU	83.1	76.6	85.1	79.72
	YOLO11n+NAU *	84.1	84.4	88.8	84.25
Ripe	YOLO11n	65.3	95.8	86.6	77.66
	YOLO11n+DWConv	60.2	95.4	81.6	73.82
	YOLO11n+ C3K2_GsConv	67.8	94.3	84.6	78.88
	YOLO11n+NAU	69.4	90.5	83.0	78.56
	YOLO11n+NAU *	80.2	87.5	87.7	83.69

Table S2 Weight distribution of the training dataset used for single-fruit weight estimation.

Type	Weight (g)	Maximum Weight (g)	Minimum weight (g)	Number of fruits
Cherry Tomato	12.01±2.89	20.33	5.31	135
Large fruit Tomato	75.02±33.15	136.38	23.71	37

Table S3 Downstream quality prediction and boundary evaluation using YOLO11n and YOLO11n-NAU masks*

Model	Vitamin C R^2	soluble solids content R^2	Firmness R^2	Acidity R^2	Protein R^2	Weight R^2	Weight RMSE	Weight MAPE
YOLO11n mask	0.87	0.73	0.88	0.92	0.89	0.79	23.09	31.08
YOLO11n-NAU* mask	0.85	0.65	0.90	0.91	0.88	0.82	21.40	29.78
Model	Boundary IoU	Boundary IoU	Boundary IoU	Boundary IoU				
YOLO11n mask	0.040	0.040	0.040	0.040				
YOLO11n-NAU* mask	0.045	0.045	0.045	0.045				

Table S4 Real sample size by cultivar and maturity stage

Trait	Cultivar	Maturity stage	Real n	Synthetic training n
Quality traits other than soluble solids	Cherry tomato	Immature	47	153
	Cherry tomato	Semi-ripe	115	375
	Cherry tomato	Ripe	120	391
	Large-fruited tomato	Immature	34	114
	Large-fruited tomato	Semi-ripe	54	176
	Large-fruited tomato	Ripe	41	134
Quality traits other than soluble solids	Total		411	1343
Soluble solids	All fruits with valid SSC measurements		247	Included in Copula augmentation comparison

Note: Real sample sizes are reported for all 411 fruits with complete cultivar and maturity records.

Table S5 Image processing configuration parameter information

Category	Configuration
CPU	AMD Ryzen 9 9950X 16-Core Processor CPU @ 3.60 GHz
GPU	Nvidia GeForce RTX 5080
Operating System	Windows11 25HZ
Deep learning framework	Pytorch2.9.0 + cuda13
Programming language	Python-3.10

Table S6 Tomato image target information

Data type	Number of image types	Category	Number of targets
Zheyinfan	649	Immature stage	4630
		Semi-ripe stage	1188
		Ripe stage	1815
Provence	1260	Immature stage	3120
		Semi-ripe stage	1060
		Ripe stage	1194
